# Case Report: Using Medtronic AP360 mechanical prosthesis in mitral valve replacement for patients with mitral insufficiency after primum atrial septal defect repair to reduce left ventricular outflow tract obstruction risk

**DOI:** 10.3389/fsurg.2022.1008444

**Published:** 2023-01-06

**Authors:** Lei Guo, Qiqi Yang, Yu Han, Haige Zhao, Liangwei Chen, Junnan Zheng, Yiming Ni

**Affiliations:** Department of Cardiovascular Surgery, The First Affiliated Hospital, College of Medicine, Zhejiang University, Hangzhou, China

**Keywords:** PASD repair, MVR, LVOTO, congenital heart disease, case report

## Abstract

**Background:**

Atrial septal defect is one of the most common congenital heart diseases in adults. Primum atrial septal defect (PASD) accounts for 4%–5% of congenital heart defects. Patients with PASD frequently suffer mitral insufficiency (MI), and thus, mitral valvuloplasty (MVP) or mitral valve replacement (MVR) is often required at the time of PASD repair. Unfortunately, recurrent unrepairable severe mitral regurgitation can develop in many patients undergoing PASD repair plus MVP in either short- or long-term after the repair surgery, requiring a re-do MVR. In those patients, the risk of left ventricular outflow tract obstruction (LVOTO) has increased.

**Case presentation:**

We present five such cases, ranging in age from 24 to 47 years, who had a PASD repair plus MVP or MVR for 14–40 years while suffering moderate to severe mitral regurgitation. Using Medtronic AP360 mechanical mitral prostheses, only one patient experienced mild LVOTO.

**Conclusions:**

The use of Medtronic AP360 mechanical mitral prostheses to perform MVR in patients with MI who had a history of PASD repair can potentially reduce the risk of LVOTO. Long-term follow-up is required to further confirm this clinical benefit associated with AP360 implantation in patients with PASD.

## Introduction

Atrial septal defect (ASD) is one of the most common congenital heart diseases in adults. Primum atrial septal defect (PASD), also known as endocardial cushion defect or partial atrioventricular (AV) septal defect, accounts for 4%–5% of congenital heart defects (1, 2). PASD is defined as a defect at the base of the interatrial septum caused by failure of the primum septum to fuse with the endocardial cushions. PASD is usually associated with defects in AV valves, especially in the anterior mitral valve leaflet, and thus, patients with PASD are mostly complicated with mitral insufficiency (MI) requiring mitral valvuloplasty (MVP) or mitral valve replacement (MVR) at the time of PASD repair. Also, recurrent severe mitral regurgitation is observed in many patients undergoing PASD repair plus MVP in either short- or long-term after the repair surgery, requiring a reoperation (3). An MVR is often required in these patients with unrepairable MI. Nevertheless, left ventricular outflow tract obstruction (LVOTO) often occurred after PASD repair in these patients, with or without MVR, leading to dyspnea, coronary insufficiency, congestive heart failure, and even death (4). Here, we present five cases of successful treatment of MI in patients with PASD repair history using Medtronic AP360 mechanical prostheses.

## Case report

### Case 1

**Figure 1:**
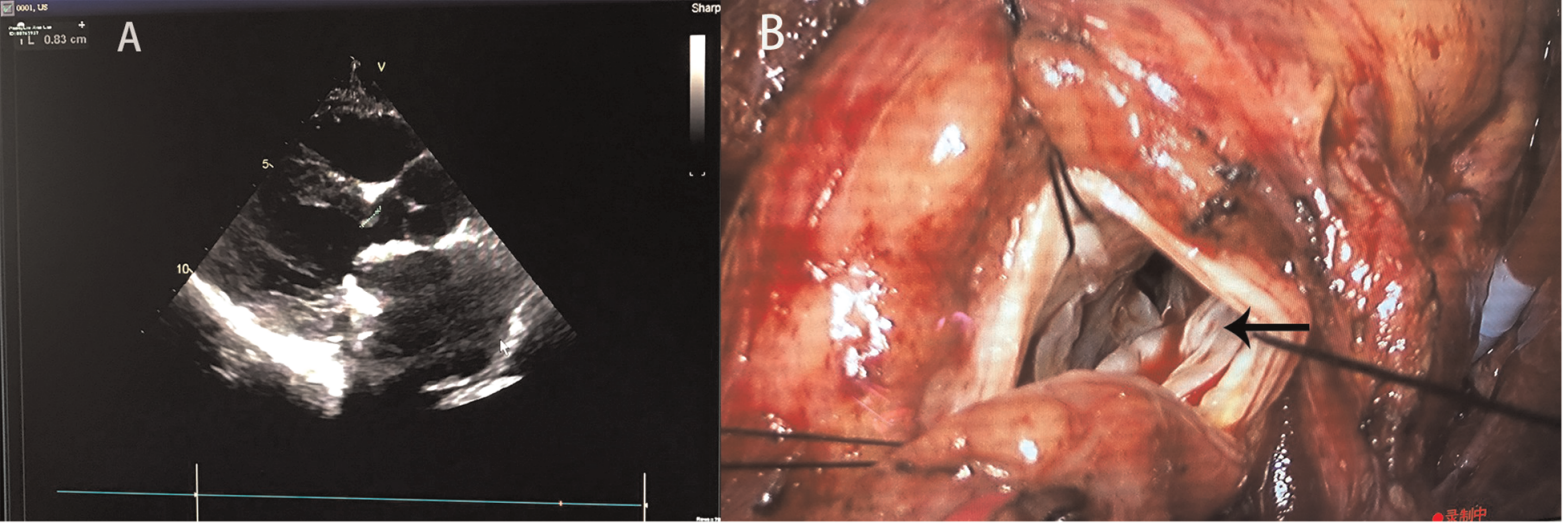
(**A**) Preoperative echocardiography showed and (**B**) intraoperative findings of Case 1. The black arrow indicates the protruding mitral annulus tissue.

A 34-year-old female was admitted to our hospital because of chest pain complaint for 2 days. She had a PASD repair followed by an MVR 5 months after the PASD repair because of MI 25 years ago. Her echocardiography showed a peak left ventricular outflow tract (LVOT) pressure gradient of 109 mmHg, left ventricular outflow peak velocity of 5.23 m/s, minimum LVOT diameter in anteroposterior dimension of 7 mm, and the thickness of muscular part of interventricular septum of 14–16 mm, causing severe LVOTO (Figure 1A). The patient underwent MVR and LVOT repair *via* median sternotomy. Intraoperative examination revealed that part of the sewing cuff of the previously implanted mitral prosthesis was covered with hyperplastic annulus fibrosus tissue, which protruded from the mitral annulus underneath the mechanical valve into the left ventricular outflow tract (Figure 1B) and that one prosthetic valve lobe failed to open completely. Thus, the hyperplastic fibrous ring on the prosthetic valve and the thickened endocardium of the ventricular septum resulted in a stenosis ring. We implanted a 22# AP360 mechanical prosthesis (Medtronic, Inc., Minneapolis, MN, USA) to replace the previous prosthetic valve. The patient's postoperative echocardiography showed that the newly implanted mechanical valve worked well without residual LVOTO.

### Case 2

Patient 2 was a 24-year-old female. She was hospitalized because of a 1-month history of palpitation, accompanied by occasional chest tightness and shortness of breath. She had PASD repair plus MVP 14 years ago. Her echocardiography at the hospital admission showed moderate to severe MI and anterior mitral valve leaflet cleft. The mitral valve was unrepairable and a 24# AP360 mechanical prosthesis implantation was performed. Mild postoperative LVOT stenosis was observed: Her postoperative echocardiography confirmed that the implanted prosthetic valve performed well and showed that her peak LVOT pressure gradient was 40 mmHg and minimum LVOT diameter in anteroposterior dimension was 8.9 mm.

### Cases 3–5

Patients 3–5 were aged from 29 to 47 years and hospitalized because of MI. They had PASD repair plus MVP 15–40 years ago. The existing mitral valve was beyond repair, and thus, MVR was performed on the patients. Medtronic AP360 mechanical prostheses of proper sizes were implanted. All three patients recovered uneventfully.

All patients underwent surgery *via* regular cardiopulmonary bypass through median sternotomy or right thoracotomy. Standard prophylactic antibiotics with intravenous second-generation cephalosporin in the right dose were given to patients with AP360 or other prostheses. After 1 year of follow-up, all five patients were still alive and in NYHA function class 1 or 2 with no major complications. Echocardiography showed no apparent abnormality (Table 1).

**Table 1:** Clinical data of five patients.

Case no.	Gender	Age	Previous surgery	Preoperative MI	Interventricular septal thickness (mm)	Preoperative gradient of LVOT (mmHg)	Preoperative peak velocity of LVOT (m/s)	Prosthesis implantation and suturing technique	Postoperative LVOTO
1	Female	34	PASD repair, MVR	Mild	14–16	109	5.23	22# AP360, non-everting suture	None
2	Female	23	PASD repair	Severe	8	<40	<3.0	24# AP360, everting suture	Mild (peak velocity 3.2 m/s, gradient 40 mmHg)
3	Male	57	PASD repair	Severe	9	<40	<3.0	24# AP360, everting suture	None
4	Female	29	PASD repair	Moderate to severe	10	<40	<3.0	24# AP360, everting suture	None
5	Female	45	PASD repair	Severe	9	<40	<3.0	26# AP360, everting suture	None

MI, mitral incompetence; LVOT, left ventricular outflow tract; LVOTO, left ventricular outflow tract obstruction; PASD, partial atrial septal defect.

## Discussion

LVOTO, which has an incidence rate of up to 6%, is one of the most common complications in patients undergoing PASD repair (5, 6). In this case series, we reported five patients with previous PASD repair history, who underwent MVR for MI in our center. All of them were implanted with a Medtronic AP360 mechanical mitral valve including one re-MVR, and only one patient developed mild postoperative LVOTO.

We used AP360 valves in these five patients because we believe that the design of AP360 could reduce the risk of LVOTO. In normal anatomy, LVOT is only a few millimeters long and the aorta is “wedged” between the mitral and tricuspid valves. However, in patients with PASD, the aortic valve is anterior and rightward but is not positioned between the two normal AV valves, which consequently changes the anteroposterior dimension of the LVOT. The should-be position of the aortic root forms an extra length of LVOT, which is absent in the heart with normal anatomy. Moreover, the deficiency of the septum makes a convexity towards the ventricular side, narrowing the LVOT. These anatomic abnormalities lead to a narrow and elongated LVOT with an abnormal outlet angle, which is described classically as the “goose neck” deformity in angiography (6). Because of the anatomic characteristics of PASD as mentioned above, performing an MVR on a PASD patient is risky. An intra-annularly placed prosthetic valve may even worsen LVOTO (7, 8).

**Figure 2:**
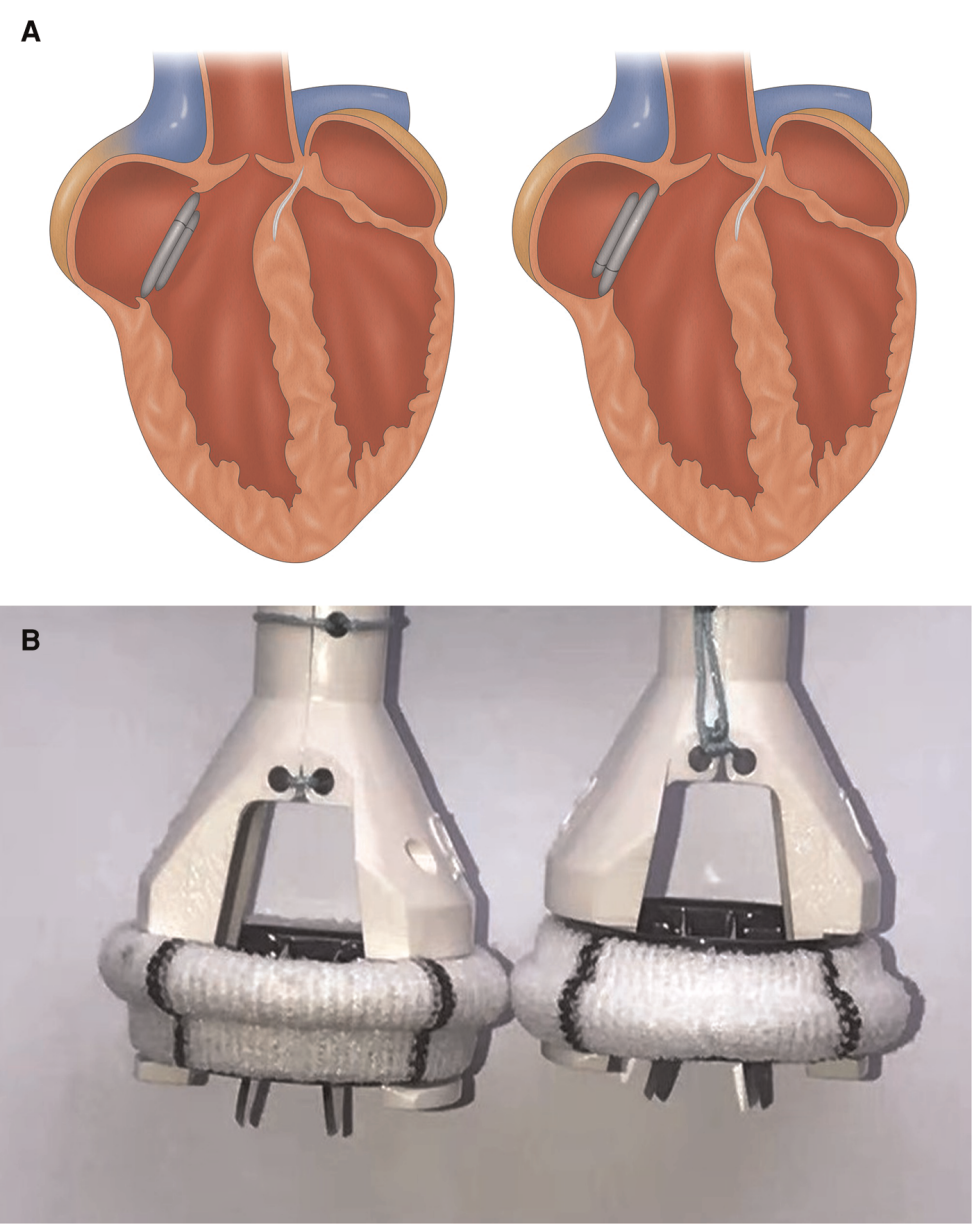
(**A**) Cartoon showing an implanted ATS mechanical mitral valve (left) and AP360 (right) after MVR, and (**B**) photos of an ATS mechanical mitral valve (left) and AP360 (right). Panel **A** shows that after the implantation, ATS valve will subside more substantially into the LVOT than an AP360, resulting in a higher risk of LVOTO. Panel **B** shows that with the sewing cuff in the same plane, the ATS is approximately 3 mm lower than the AP360. MVR, mitral valve replacement; LVOT, left ventricular outflow tract; LVOTO, left ventricular outflow tract obstruction.

Medtronic AP360 mechanical prosthesis has unique advantages. Thanks to its special design which looks like an inverted ATS mechanical prosthesis, it locates mostly on the atrial side (Figure 2B). Combined with supra-annular placement, the AP360 prosthesis can be lifted by several millimeters and thus reduce the influence on the LVOT to the minimum. While intra-annular placement makes the main part of a prosthetic valve, such as ATS mechanical prosthesis, locate on the ventricular side, narrowing the LVOT even further (Figure 2A).

Besides, a prosthetic valve with pannus and hyperplastic annulus fibrous tissue can partially block the LVOT and thus increase the risk of LVOTO. In Case 1 of this report, we found that part of the sewing cuff of the previously implanted prosthetic valve was covered with hyperplastic annulus fibrous tissue protruding into the LVOT. The sewing cuff of the AP360 mechanical prosthesis, which is not in the same horizontal plane as the prosthetic valve's opening and different from other prosthetic valves in terms of positioning, can avoid hyperplastic annulus fibrous tissue formation. Thus, the potential risk of LVOTO associated with AP360 prosthesis implantation can be reduced.

Notably, the Carbomedics mechanical mitral prosthesis can be squeezed by the scarred annulus and thus sink into the ventricular side (normally pushed to the atrial side due to pressure difference) and worsen LVOTO because of its flexible and longitudinal symmetrical sewing cuff. Although other types of mechanical valves with a similar design as the ATS prosthetic valves maybe not be as bad as the Carbomedics valves in terms of the risk of LVOTO, could still sink to the LV to some degree. Implantation of Medtronic AP360 prosthesis using a non-everting suture can lift the prosthesis to the left atrium (LA) and avoid this situation.

We had encountered a case showing Carbomedics mitral valve implantation associated with LVOTO. A 40-year-old female was diagnosed with primum ASD, anterior mitral valve leaflet cleft, and moderate tricuspid regurgitation. She underwent tricuspid valvuloplasty and PASD repair. However, her mitral valve was beyond repair and thus a 27# Carbomedics mechanical mitral valve was then implanted. Her postoperative TEE showed the well-performed implanted prosthetic mitral valve, a peak LVOT pressure gradient of 34 mmHg, and left ventricular outflow peak velocity of 2.9 m/s. Her postoperative 3-month follow-up echocardiography showed moderate LVOT stenosis with a peak LVOT pressure gradient of 73 mmHg, left ventricular outflow peak velocity of 4.2 m/s, minimum LVOT diameter in an anteroposterior dimension of 13 mm, and an interventricular septum thickness of 10 mm. Postoperative 18-month follow-up echocardiography showed severe LVOT stenosis with a peak LVOT pressure gradient of 92 mmHg, left ventricular outflow peak velocity of 4.8 m/s, minimum LVOT diameter in an anteroposterior dimension of 6.6 mm and an interventricular septum thickness of 12 mm. The patient refused a re-MVR. Similarly, the Mayo Clinic had reported a case of acute LVOTO after Carbomedics MVR (9), and they used a non-everting suture to solve this problem, which coincided with us on this point.

Actually, LVOTO was more common in patients who had to have their MVR re-done. We compared the difference in LVOT dimension and LVOT gradients of patients who underwent re-do MVR for different reasons with various mechanical mitral prostheses, as shown in Table 2. For St. Jude, Carbomedics and ATS mitral valve, there is no significant change in LVOT dimension and max LVOT gradients between pre- and post-surgery, while for AP360 group, the LVOT dimension of post-surgery is larger than pre-surgery and the max LVOT gradients is significantly reduced after surgery. The sample capacity was not large enough and the fact that a part of the patients of the AP360 group was already complicated with LVOTO before surgery might partially contribute to the result. However, according to our experience, we still believe choosing an AP360 prosthesis with a proper size matching the patient's anatomic structure is critical for good clinical outcomes.

**Table 2:** Mitral prosthesis and LVOT dimension in re-do MVR patients.

Types of mitral prostheses	Pre-surgery LVOT dimension (mm)	Post-surgery LVOT dimension (mm)	*P*-value	Pre-surgery max LVOT gradients (mmHg)	Post-surgery max LVOT gradients (mmHg)	*P*-value
Medtronic ATS (*n* = 11)	18.46 ± 1.56	18.00 ± 3.22	NS	10.75 ± 5.76	9.93 ± 4.37	NS
Medtronic AP360 (*n* = 5)	15.41 ± 3.43	17.68 ± 2.08	<0.001	61 ± 53.98	19.28 ± 10.15	<0.001
Carbomedics (*n* = 9)	17.67 ± 1.66	17.22 ± 1.79	NS	5.76 ± 3.64	6.31 ± 1.24	NS
St. Jude (*n* = 12)	17.50 ± 1.57	17.42 ± 1.52	0.018	5.38 ± 2.08	5.18 ± 2.33	NS

LVOT, left ventricular outflow tract; NS, not significant; MVR, mitral valve replacement.

On the other hand, an oversized AP360 may cause valve dysfunction. One of the lobes of an oversized prosthesis could be blocked by the thickened interventricular septum, leading to valve dysfunction. Aggressive implantation of an oversized prosthetic valve to avoid mitral prosthesis-patient mismatch, which remains to be controversial regarding the patient outcome, thus is not recommended (10).

Some pediatric cardiac surgeons suggested that the chimney technique could be used to avoid LVOTO in young patients undergoing MVR (11). The Chimney technique is to suture a several-millimeter-long tubular dacron graft to the sewing cuff of a prosthetic valve and then suture the graft to the native mitral annulus, forming a composite graft floating in the LA like a “chimney”. Although the chimney technique showed promising LVOTO prevention in children, the long-term effect is still unknown in patients with PASD.

## Conclusion

Usage of Medtronic AP360 mechanical mitral prostheses to perform MVR in patients with MI who had a PASD repair history can potentially reduce the risk of LVOTO. Long-term follow-up is required to further confirm this clinical benefit associated with AP360 implantation in patients with PASD.

## Data Availability

The original contributions presented in the study are included in the article/Supplementary Material, further inquiries can be directed to the corresponding author/s.
